# Reply to Kaykov et al. Comment on “Chowdhury et al. Impact of Molecular Testing on Surgical Decision-Making in Indeterminate Thyroid Nodules: A Systematic Review and Meta-Analysis of Recent Advancements. *Cancers* 2025, *17*, 1156”

**DOI:** 10.3390/cancers18142307

**Published:** 2026-07-17

**Authors:** Raisa Chowdhury, Jessica Hier, Kayla E. Payne, Mawaddah Abdulhaleem, Orr Dimitstein, Netanel Eisenbach, Véronique-Isabelle Forest, Richard J. Payne

**Affiliations:** 1 Faculty of Medicine and Health Sciences, McGill University, Montreal, QC H3A 0G4, Canada; 2 Department of Otolaryngology-Head and Neck Surgery, McGill University Health Center, Montreal, QC H4A 0B1, Canada; 3 Department of Otolaryngology-Head and Neck Surgery, Jewish General Hospital, McGill University, Montreal, QC H3T 1E2, Canada; 4 Faculty of Arts, McGill University, Montreal, QC H3A 0G4, Canada; 5 Department of Otolaryngology Head and Neck Surgery, Thyroid and Parathyroid Surgery Division, Dr. Suliman Alhabib Hospital, Jeddah 22245, Saudi Arabia

We thank the commenter for their interest in our systematic review and meta-analysis evaluating the impact of molecular testing on surgical decision-making in indeterminate thyroid nodules.

Several points raised in the comment relate to the originally published version of the manuscript [1,2]. Following internal review, we had identified that a small number of conference abstracts had been inadvertently included despite our stated exclusion criteria. To ensure methodological consistency and transparency, the dataset was reassessed and the meta-analysis repeated after removal of these records.

After exclusion of these abstracts, the dataset changed from 31 studies (4464 nodules) to 26 studies (3950 nodules). Importantly, the recalculated pooled estimates demonstrated only modest numerical adjustments and did not materially change the interpretation or overall conclusions of the study. For example, pooled surgical avoidance rates remained approximately 64% for ThyroSeq V3, 59–60% for Afirma GSC, and 66–67% for ThyGenX/ThyraMIR, maintaining the same overall trends reported in the original analysis.

Study screening and data extraction were performed independently by two reviewers using Covidence systematic review software in accordance with the PRISMA methodology, with discrepancies resolved by consensus review. The inclusion and exclusion criteria specified the evaluation of molecular testing platforms in Bethesda III/IV indeterminate nodules. All studies underwent full-text eligibility assessment according to the predefined inclusion and exclusion criteria.

The commenter also references individual studies reporting surgical avoidance rates for specific platforms. As expected in systematic reviews synthesizing heterogeneous real-world datasets, variations in patient populations, institutional practices, follow-up duration, and study design may lead to differences between pooled estimates and individual institutional cohorts. Our analysis reflects aggregated evidence across multiple institutions and study designs; therefore, pooled estimates derived from heterogeneous multicenter datasets may differ from results reported in individual platform-specific cohorts.

Importantly, even after the correction of the dataset, the overall conclusions of the study remain unchanged: molecular testing platforms are associated with substantial reductions in diagnostic thyroid surgery for indeterminate nodules, with pooled surgical avoidance rates ranging from approximately 50% to 67% across platforms, and with limited representation for some platforms, warranting cautious interpretation.

We appreciate the opportunity to clarify these points and confirm that the updated analysis reflects the most accurate and transparent synthesis of the currently available literature.

## Data Availability

No new data were created or analyzed in this study. Data sharing is not applicable to this article.
